# Lung cancer tumor immune microenvironment: analyzing immune escape mechanisms and exploring emerging therapeutic targets

**DOI:** 10.3389/fimmu.2025.1597686

**Published:** 2025-09-25

**Authors:** Zhen Wang, Honglei Guo, Yanqi Song, Aidi Wang, Yuting Yan, Lin Ma, Baoshan Liu

**Affiliations:** ^1^ Graduate School, Tianjin University of Traditional Chinese Medicine, Tianjin, China; ^2^ Tianjin Medical University General Hospital, Tianjin, China

**Keywords:** lung cancer, tumor immune microenvironment, immune escape, therapeutic targets, mechanism studies

## Abstract

Lung cancer is the most common malignant tumor in the world. Presently, there are still problems, including a high recurrence rate, resistance, and serious toxic side effects, even if conventional treatments like chemotherapy, radiotherapy, and targeted therapy have somewhat improved patient survival. Even though immune checkpoint inhibitors that target programmed cell death-1/programmed cell death ligand 1 have fundamentally altered the therapeutic paradigm, the core mechanism is strongly linked to tumor immune escape, and some patients continue to have poor response rates or treatment resistance. The mechanisms of immune escape in the immunological microenvironment of lung cancer, involving metabolic reprogramming, overexpression of immune checkpoint molecules, and abnormalities in antigen presentation, are systematically summarized in this review. The article also sums up new therapeutic targets and promising clinical trials. The goal is to provide a solid theoretical foundation for further research into the immune escape mechanism, the creation of new immunotherapeutic targets, and personalized therapeutic strategies.

## 1 Introduction

Lung cancer has placed a significant strain on healthcare systems around the world due to its high incidence and fatality rates, ranking it at the top of the list of malignant tumors (1). Patients with early-stage lung cancer have the opportunity to choose surgery, while patients with advanced or metastatic lung cancer are often treated with systemic therapy or combined with local radiotherapy (2). Radiation therapy, chemotherapy, and targeted therapy currently dominate the standard treatment options for lung cancer. While these treatments have extended progression-free survival (PFS) for patients to some degree, numerous individuals continue to encounter issues of post-treatment recurrence or resistance, and the adverse side effects linked to the treatments seriously hamper patients’ quality of life (3). The advent of immune checkpoint inhibitors (ICIs), represented by programmed cell death-1/programmed cell death ligand 1 (PD-1/PD-L1), has dramatically changed lung cancer treatment (4). Nevertheless, the study indicated that only a limited number of patients had long-term responses to ICIs, while the majority were unable to overcome the issue of resistance (5). There is increasing evidence that immune escape is a critical factor contributing to immunotherapy resistance and disease progression in lung cancer patients (6–9). To improve the effectiveness of immunotherapy, reverse medication resistance, and enhance the survival of patients with lung cancer, it is crucial to comprehensively study the mechanisms of immune escape. As basic and clinical trials keep moving forward, so do studies on immunotherapeutic targets for lung cancer. These studies are growing and becoming more in-depth, looking at everything from traditional immune checkpoints to the discovery of new targets and from combination therapy strategies to the investigation of personalized treatment protocols, all of which give lung cancer patients more hope.

## 2 Overview of the immune microenvironment in lung cancer

According to studies, the tumor microenvironment has a significant role in the proliferation and occurrence of malignant tumors, mostly by accelerating tumor cell invasion and metastasis, stimulating the production of new blood vessels, and encouraging immune escape (10). The tumor immune microenvironment mainly refers to the microenvironment dominated by immune cells. T cells and B cells comprise most of the adaptive immune system, while tumor-associated macrophages, natural killer cells, regulatory T cells, and dendritic cells constitute innate immune cells (11, 12). During the progression of lung cancer, cells from the immune system, stromal cells, and the extracellular matrix interact to create a complex ecological setting known as the immune microenvironment (13). Based on recent research, tumor-derived exosomes and fibroblastic reticular cells also contribute to the emergence of lung cancer immune microenvironment (14, 15). The lung cancer immune microenvironment can usually be divided into two categories: immune-activated and immune-suppressed. Immunosuppressive cells and molecules like Tregs, M2-type tumor-associated macrophages (TAMs), and transforming growth factor-β (TGF-β) suppress anti-tumor immune responses. In contrast, immune-activating cells and pro-inflammatory cytokines, including CD8+ T cells and γ-interferon, promote anti-tumor immunity (16, 17).

In early-stage lung cancer, the key feature is immune infiltration, characterized by a high enrichment of CD8+ tissue-resident memory T cells (CD103+CD69+TRM), which predominantly aggregate at the tumor parenchyma–stroma interface. Localized advanced or locally advanced lung cancer may manifest as immune cell reprogramming, with immune suppression dominating, a reduction in CD8+ TRM, and TAM predominantly of the M2 type (18, 19). In advanced or metastatic lung cancer, characteristics may include immune exhaustion and heterogeneity, manifested by an increased proportion of exhausted T cells in peripheral blood and metastatic lesions (20).

The “3E” model, put forth by Dunn et al. in 2002, provides a detailed description of the three stages of interactions between cancers and the immune system: immunological escape, immune equilibrium, and immune elimination (21). The immune system plays a crucial role in preventing tumors from forming in the early stages of lung cancer. During this time, any remaining tumor cells are carefully monitored and do not contribute to tumor progression. However, as time goes on, some tumor cells manage to escape the immune system’s control mechanisms through either genetic mutations or epigenetic reprogramming. At this point in the progression of the disease, the cells undergo rapid growth and proliferation. This indicates that immune escape is the main cause of disease progression or resistance to immunotherapy in patients with advanced lung cancer. In 2024, Galassi et al. introduced a comprehensive “3C” paradigm that methodically elucidates the methods by which tumor cells achieve immune evasion, identifying three primary strategies: camouflage, coercion, and cytoprotection (22). These mechanisms not only involve alterations at the molecular level but are also closely related to the sophistication of the tumor microenvironment. All things considered, tumor cells in the tumor microenvironment utilize a variety of strategies to elude the immune system’s attack, which results in inadequate immunotherapy effectiveness and the issue of resistance (23).

## 3 The research progress on the mechanisms of immune escape in lung cancer

### 3.1 Internal escape mechanisms

#### 3.1.1 Defect in antigen presentation

Human leukocyte antigen class I (HLA-I) molecules play a key role in tumor antigen presentation and immune surveillance. Tumor cells may be unable to effectively present antigens due to the loss or downregulation of HLA-I expression, which could prevent the immune system from identifying and attacking them (24). Research has indicated (25) that HLA-I heterozygous deletions are prevalent in advanced or metastatic lung cancer, particularly in tumors with moderate tumor mutational burden (TMB) levels. However, the incidence decreased when the TMB was over 30 mutations, which indicates that highly mutated tumors may need to evade immune surveillance through other mechanisms. According to Montesion et al., the loss of HLA-I heterozygosity reduced TMB’s predictive power as an indicator of ICI efficiency (25). This suggests that TMB and HLA-I heterozygosity deletion together could be a more precise method to predict the effectiveness of immunotherapy in patients with lung cancer.

Furthermore, the heterogeneous expression of tumor neoantigens is also an essential mechanism for immune escape in lung cancer (26). When tumor cells undergo mutations, they produce neoantigens, which are unique antigens that the immune system may identify and use to mount an attack against the tumor. However, neoantigens’ immunogenicity is determined by their co-expression and clonality, and heterogeneous expression may cause tumor cells to selectively express particular neoantigens, escaping immune surveillance. This also partly explains the poor response of some patients to immunotherapy. By accurately identifying and targeting highly immunogenic neoantigens, this finding offers a theoretical foundation for the creation of tailored neoantigen vaccines, which could boost immunotherapy effectiveness and decrease immune escape in the future (26, 27).

#### 3.1.2 Overexpression of immune checkpoint molecules

The abnormal expression of immune checkpoint molecules is one of the core mechanisms of immune escape in lung cancer. For example, individuals with epidermal growth factor receptor (EGFR)-mutated non-small cell lung cancer (NSCLC) commonly possess an immunosuppressive microenvironment, resulting in poorer response rates to immunotherapy (28). In NSCLC patients harboring EGFR mutations, the expression level of the transmembrane protein CD47, an innate immunological checkpoint, was markedly elevated (29). By activating the downstream extracellularly regulated protein kinases and serine/threonine kinase pathways, which are both linked to the CD47 promoter area and hence strengthen CD47 expression, it was shown that EGFR mutations upregulate the transcription factors c-Myc and nuclear factor kappa-light-chain-enhancer of activated B cells. When highly expressed CD47 is combined with signal regulatory protein α on the surface of macrophages, it can inhibit the phagocytic ability of macrophages and help tumor cells evade immune surveillance (30).

Additionally, histone lactylation has been proven to mediate tumor immune escape and remodel the tumor microenvironment (31). Zhang et al. demonstrated that undesirable patient outcomes are linked to increased levels of pan-lysine lactylation and histone H3 lysine 18 lactylation (H3K18la) in NSCLC (32). H3K18la can directly bind to the CD274 promoter, enhancing the overexpression of PD-L1 and the immune escape ability of tumor cells. It can also directly boost the transcription of POM121 transmembrane nucleoporin, which improves the nuclear transport of MYC. The reversal of immune escape is achievable by metabolic reprogramming and anticancer immunotherapy, offering a novel perspective for investigating the immune escape mechanisms in lung cancer.

Acetate is a plentiful short-chain fatty acid in tumor cells and can participate in cellular metabolic processes as a metabolic substrate (33). Acetate-derived acetyl coenzyme A was found to increase c-Myc expression through the dihydrolipoamide *S*-acetyltransferase-mediated acetylation of lysine at position 148 of c-Myc, which is correlated with low overall survival in NSCLC patients (34). Acetylated c-Myc further stabilizes its expression by recruiting the deubiquitinase USP10. The higher stability and expression of c-Myc directly cause PD-L1 expression to be upregulated, which in turn reduces the activation of CD8+ T cells and so causes tumor cells to evade immune tracking. Furthermore, by altering the tumor microenvironment, acetate can produce an immunosuppressive milieu by decreasing immune-activated cells and increasing immune-suppressed cells.

#### 3.1.3 Tumor metabolic reprogramming

Another important way that lung cancer cells evade the immune system is by metabolic reprogramming of tumors (35). Metabolic reprogramming and mitochondrial malfunction in tumor-infiltrating lymphocytes can hamper anti-tumor immune responses, and mitochondria are required for immune cell metabolic reprogramming (36). According to earlier studies (37, 38), a genetic mutation in mitochondrial DNA (mtDNA) contributes to the development and spread of several malignant tumors. Ikeda et al. found that mtDNA mutations can lead to abnormal mitochondrial function, contributing to the transfer of mitochondria from tumor cells to T cells, resulting in T-cell dysfunction (39). Mitochondria are the powerhouses of cells, and mtDNA mutations cause mitochondrial dysfunction, which in turn affects T-cell energy metabolism, preventing them from meeting their energy needs during immune responses. Mitochondrial translocation also leads to increased oxidative stress within T cells, which then triggers T-cell senescence and apoptosis. Apoptotic T cells directly lose their immune function. In addition, mitochondrial dysfunction also impairs T-cell activation and effector function. All of these issues collectively weaken the anti-tumor capacity of T cells, allowing tumor cells to evade T cell-mediated immune surveillance. Furthermore, mtDNA mutations induce an immunosuppressive milieu, encourage glycolysis, and impair oxidative phosphorylation. The mutation of mtDNA in lung cancer tissues is an adverse factor for patients with decreased response rates to immunotherapy. Overall, this study sheds light on a new way that tumor cells use mitochondrial metastasis to get around the immune system, which could be beneficial for creating immunotherapies for lung cancer.

R-loop is a special three-stranded nucleic acid structure, and its abnormality is also closely correlated with lung cancer immune escape (40, 41). Zhang et al. constructed the R-loop scoring model using single-cell ribonucleic acid (RNA) sequencing (42). Low R-loop score lung cancer cells were found to substantially express tumor progression-related genes and concurrently increase immunosuppressive factors, generating a strong immunosuppressive milieu supporting the proliferation and immune escape of lung cancer cells. Moreover, these cells exhibited a pattern of metabolic reprogramming associated with T-cell depletion, promoting drug resistance. The R-loop score model provides a promising marker for predicting response to immunotherapy in lung cancer patients.

In addition, a research team recently discovered that R-loops play an interesting paradoxical role in cancer (43). Excessive R-loop formation may lead to genomic instability, thereby promoting tumor initiation and progression, while R-loop-mediated innate immune responses via the cyclic GMP–AMP synthase-stimulator of the interferon gene pathway can trigger anti-tumor immunity. In the tumor microenvironment, R-loop-mediated genomic instability triggers an inflammatory signaling cascade that affects tumor cells and their surrounding tumor microenvironment. High R-loop scores are associated with enhanced chemokine and co-stimulatory molecule expression, which facilitates the recruitment and activation of immune cells, while tumor cells with low R-loop scores exhibit downregulation of tumor-associated antigen and MHC molecule expression and upregulation of immune inhibitory factor expression, promoting immune escape. Together, these findings provide a theoretical basis for R-loop-based therapeutic strategies and may improve the clinical outcomes of lung cancer patients.

#### 3.1.4 Abnormal regulation of ribosomal P-stalk

The ribosome is a central machine for protein synthesis (44). A GTPase-associated subunit on ribosomes called P-stalk tends to preferentially translate mRNAs linked to immune surveillance and cytokine responses. Researchers at the Netherlands Cancer Institute have found that tumor cells suppress p-stalk expression and reduce antigen presentation to dodge detection (45). The reduction of P-stalk significantly decreased the expression of HLA-I molecules and hampered the ability of tumor cells to be recognized and killed by CD8+ T cells. Therefore, tumor cells may elude immune monitoring or contribute to immunotherapy resistance by downregulating P-stalk proteins or inhibiting P-stalk ribosomes.

### 3.2 Abnormal activation of the immune checkpoint pathways

A significant source of immunological escape is the abnormal activation of immune checkpoint pathways, which can impact the immune milieu through several methods (46, 47). Otubain-2 (OTUB2) is a deubiquitinating enzyme that is aberrantly expressed in several cancer types, and its activation is involved in the progression and metastasis of tumors (48). By facilitating tumor immune evasion via the PD-1/PD-L1 pathway, the researchers discovered that OTUB2, which is produced by tumor cells, functions as a negative regulator of anti-tumor immunity (49). OTUB2 expression levels are correlated with higher PD-L1 and lower CD8+ T-cell profiles in NSCLC patient samples. The PD-L1 protein is then much less expressed on the surface of tumor cells when OTUB2 is absent, which increases the tumor cells’ susceptibility to CD8+ T cells and intra-tumor T-cell infiltration and mediates the tumor cells’ ability to be eliminated. Furthermore, the application of the OTUB2 inhibitor may diminish its deubiquitinase activity, effectively lower PD-L1 expression in tumor cells, and impede tumor cell proliferation, thereby enhancing anti-tumor immune efficacy.

According to a study, peroxiredoxin-2 (PRDX2) is strongly expressed in lung cancer and is directly linked to survival, along with the development of tumor cells (50). Patients with higher expression levels of PRDX2 have a worse prognosis. Galactose lectin-9 (Galectin-9) is mostly found in antigen presentation, activated by interferon-β and interferon-γ in tumor cells, and interacts with immune cell surface receptors to decrease immunity. Histone deacetylase 3 (HDAC3) serves as a downstream effector of PRDX2 to upregulate Galectin-9 expression (51). Dong et al. established that PRDX2 modulates Galectin-9 expression through the phosphorylation of HDAC3. Galectin-9 binds to inhibitory receptors on the surface of T cells, therefore impairing the cytotoxic activity of T cells and facilitating immune evasion (52). The sensitivity of lung adenocarcinoma cells to T-cell death was considerably increased by PRDX2 expression suppression, according to cellular tests. PRDX2 inhibitor Conoidin A has demonstrated the ability to reduce tumor burden and enhance T-cell infiltration in mouse models. In the future, it may be considered for use in conjunction with existing immune checkpoint inhibitors to enhance anti-tumor immune responses. These studies lay the groundwork for future inhibitors of the immune checkpoint pathway to be developed.

### 3.3 Remodeling the tumor immune microenvironment

The remodeling of the tumor immune microenvironment is the primary component facilitating lung cancer evasion (53). TAMs play a pivotal role in the development, progression, and immune escape of lung cancer. By interacting with triggering receptors expressed on myeloid cells 2 (TREM2), Galectin-3 inhibited TREM2-mediated phagocytosis and converted TREM2+ macrophages to immunosuppressive TAMs. Anti-tumor immune responses were suppressed, and tumor cells were able to evade immune surveillance, given that this change reduced the antigen presentation and co-stimulation of TAMs. The researchers tested the effects of Galectin-3 and TREM2 on lung cancer. The findings revealed that inhibiting the activities of Galectin-3 and TREM2 considerably slowed the advancement of lung cancer, modified the tumor immune microenvironment, and significantly boosted the infiltration of CD8+ T cells and natural killer cells to strengthen anti-tumor immune responses (54).

Moreover, the inositol-requiring enzyme 1α (IRE1α)-X-box binding protein 1 signaling pathway also plays a vital role in lung cancer (55). The researchers discovered (56) that turning on IRE1α not only assisted lung cancer cells to grow and survive but also caused immune-suppressing cells to accumulate, which changed the environment around the tumor and weakened the immune response against it. The deletion or knock-out of the IRE1α gene can significantly restrain the growth of lung cancer cells. The expression of immune-related genes, particularly those on interferon response, innate immunity, and adaptive immunity, was markedly upregulated in lung cancer when IRE1α was deleted. It was suggested that inhibiting the IRE1α signaling pathway could modify the tumor immune microenvironment and boost anti-tumor immunity.

### 3.4 Abnormal regulation of cytokines and chemokines

Furthermore, lung cancer immune escape is also influenced by chemokines and cytokines (57, 58). Heim et al. discovered that interleukin-9 (IL-9) receptor-positive tumor cells and tumor-infiltrating lymphocytes are among the cells that respond to IL-9 (59). IL-9 can prevent the apoptosis of lung cancer cells and promote their growth. By altering the function of the T-cell subpopulation, IL-9 can both suppress the anti-tumor immune response and facilitate the immunological escape of NSCLC. This provides more evidence that IL-9 pathway truncation or antibody targeting IL-9 could be effective immunotherapy treatments for lung cancer.

Chemokine ligand 4 (CCL4) is an inflammatory chemokine that is typically absent or present at minimal levels in normal tissues, yet it is significantly upregulated in various malignant tumors, including lung cancer (60). By interacting with its receptor C-C chemokine receptor type 5, CCL4 can decrease anti-tumor immune responses by recruiting cells that release immunosuppressive molecules, including IL-10 and TGF-β (61). T-cell exhaustion and elevated CCL4 expression are tightly associated with the lung cancer microenvironment. Moreover, elevated CCL4 expression in lung cancer correlated with the overexpression of immune checkpoint markers, indicating potential collaboration with the PD-1/PD-L1 pathway to enhance immune evasion (62). This suggests that in patients with NSCLC, CCL4 may be an attractive target for immunotherapy.

### 3.5 Other potential mechanisms

In addition to the aforementioned mechanisms involved in lung cancer immune escape, there are also some potential receptors that may participate in the process of lung cancer immune escape through other mechanisms. For example, the miR-23a/27a/24–2 cluster regulates the expression of immune checkpoints, EMSY Transcriptional Repressor (EMSY) may influence the immunogenicity of tumor cells, and the activation of purinergic 2X7 (P2X7) receptors affects the activity of immune cells. All of these effects can influence the process of immune escape in lung cancer. These findings provide new targets and strategies for immunotherapy in lung cancer.

The miR-23a/27a/24–2 cluster is a group of microRNAs that allows tumor cells to keep out the immune system. The investigators discovered (63) that miRNAs in the miR-23a/27a/24–2 cluster upregulate PD-L1 expression by targeting Cbl proto-oncogene B, facilitating immune escape in NSCLC. By targeting transcription factors linked to microphthalmia, the cluster increased the level of eukaryotic initiation factor 3B, which further enhanced immune evasion by downregulating MHC-I expression. The expression of the miR-23a/27a/24–2 cluster was associated with the activation of the Wnt/β-catenin signaling pathway. Furthermore, studies have shown that the high expression of miR-23a/27a/24–2 clusters blocked CD8+ T-cell infiltration into tumor tissues and decreased the sensitivity of lung cancer cells to PD-L1 therapy. This work demonstrated that the miR-23a/27a/24–2 clusters play a crucial role in understanding immune escape mechanisms and resistance to immune checkpoint inhibitors, thereby setting a basis for future investigation.

EMSY is a transcriptional repressive factor and breast cancer susceptibility gene 2 inhibitory protein. Numerous malignancies, such as NSCLC, breast cancer, and ovarian cancer, have been correlated with the overexpression or gene amplification of EMSY, and the amplification of EMSY is closely linked to a negative outcome for patients (64). Kelch-like ECH-associated protein 1 (KEAP1) is frequently mutated in NSCLC, and tumors with KEAP1 mutations typically display characteristics of immune evasion. Marzio et al. found (65) that KEAP1 deletion results in EMSY protein accumulation, which raises the burden of tumor mutations and induces genomic instability in tumor cells. Also, too much EMSY stops immune cells from entering and activating by blocking type I interferon responses. The stop innate immune signals and help tumor cells evade the immune system. Likewise, by controlling immune cells in the surrounding environment, the buildup of EMSY not only impacts the tumor cells but also encourages tumor growth.

The P2X7 receptor is an ion channel activated by extracellular adenosine triphosphate, which is widely expressed in immune cells and tumor cells (66). It is critical for the immune response, the tumor microenvironment, and the proliferation of tumors. On tumor cells, the P2X7 receptor is believed to encourage tumor growth and metastasis (67). However, the P2X7 receptors have the opposite function to modulate anti-tumor immune responses. According to one study (68), the following factors may be related to the way that P2X7 receptors promote immune escape in lung cancer: 1) the activation of the P2X7 receptor can affect immune cell infiltration and function. For instance, by altering macrophage polarization and encouraging the accumulation of TAMs, it can inhibit anti-tumor immunity (2). By altering the expression of immune checkpoint molecules like PD-L1, the activation of P2X7 receptors may encourage lung cancer cells to elude immune monitoring (3). It is possible that the non-porous functional version of the P2X7 receptor regulates extracellular ATP levels or the release of its metabolites, which in turn affects immune cell activation indirectly (4). The P2X7 receptor may promote immune escape by modulating the activity of immune cells. This study demonstrates that P2X7 receptors may modulate immune responses inside the tumor microenvironment via multiple techniques, facilitating the evasion of lung cancer cells from immune system attacks.

However, P2X7 receptors exert different effects in different tumor types (69). For example, researchers have found that P2X7 receptor agonists such as BzATP exhibit anti-tumor effects in melanoma models, while in lung adenocarcinoma, the P2X7 receptor inhibitor A740003 can reduce tumor metastasis and spread. Given the complex dual role of the P2X7 receptor in tumor cells and immune cells, its specific mechanisms of action in different tumor types and microenvironments require further investigation (68). Therefore, when applying it clinically, it is necessary to consider its dual effects on immune cells and tumor cells to avoid potential side effects. Future studies should further explore the specific mechanism of action of P2X7 receptors in different lung cancer types and develop precision therapeutic strategies targeting P2X7 receptors.

**Figure 1:**
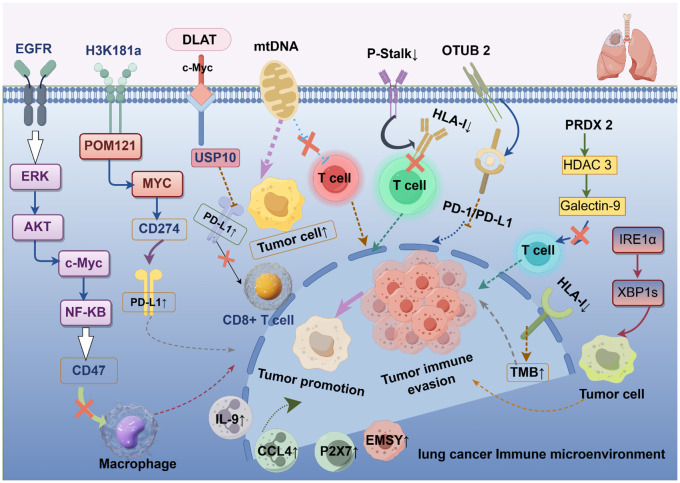
Mechanisms of immune escape in lung cancer. EGFR, epidermal growth factor receptor; ERK, extracellularly regulated protein kinases; AKT, protein kinase B; c-Myc, myelocytomatosis oncogene; NK-κB, nuclear factor kappa-light-chain-enhancer of activated B cells; H3K18la, histone H3 lysine 18 lactylation; POM121, POM121 transmembrane nucleoporin; PD-1/PD-L1, programmed cell death-1/programmed cell death ligand 1; DLAT, dihydrolipoamide *S*-acetyltransferase; USP10, ubiquitin specific peptidase 10; mtDNA, mitochondrial DNA; HLA-I, human leukocyte antigen class I; OTUB2, otubain-2; PRDX2, peroxiredoxin-2; HDAC3, histone deacetylase 3; Galectin-9, galactose lectin-9; IRE1α, inositol-requiring enzyme 1α; TMB, tumor mutational burden; IL-9, interleukin-9; CCL4, chemokine ligand 4; EMSY, EMSY Transcriptional Repressor; P2X7, purinergic 2X7.

In summary, lung cancer immune escape involves multiple mechanisms of action, which indicates the complexity of the lung cancer immune microenvironment. However, many mechanisms have not yet been systematically studied, and further validation is needed in the future to clarify their mechanisms of action in order to develop more therapeutic targets against different mechanisms of action. **Table 1** shows the other potential mechanisms of immune escape in lung cancer. **Figure 1** shows the mechanisms of immune escape in lung cancer.

**Table 1:** Other potential mechanisms of immune escape in lung cancer.

References	Genes, proteins, pathways, or axes	Mechanisms of action	Consequences of action
(70)	SKIL	Upregulating the TAZ/autophagy axis	Inhibited STING pathway, decreased chemokines (CXCL10, CCL5, and IFN-β), and T-cell infiltration and activation.
(71)	CD147	Negative regulation of CD8+ T cells	Undermined the effector function of CD8+ T cells by downregulating cytotoxic molecule levels and repressed cytotoxic genes by interfering with the functions of T-bet and Runx3.
(72)	A20 (TNFAIP3)	Loss of A20 activates the TBK1 and STAT1 signaling pathways	Upregulated PD-L1, altered the cytokine expression profile in the tumor microenvironment, and reduced CD8+ T-cell infiltration.
(73)	M3G	Activated the TLR4 signaling pathway and promoted the release of inflammatory factors	Upregulated PD-L1 expression and significantly attenuated T cell-mediated anti-tumor immune responses.
(74)	Notch1/TAZ axis	Promoted metabolic reprogramming of tumor cells and upregulated mRNA and protein expression of PD-L1	Remodeled the tumor immune microenvironment, inhibited T-cell activity, and increased infiltration of immunosuppressive cells.
(75)	TDEs	High expression of PD-L2 and binding to PD-1 on the surface of T cells impairs anti-tumor immune responses	Increased proportion of Treg cells and decreased infiltration of cytotoxic CD8+ T cells.
(76)	LINC01140	Protection of PD-L1 and c-Myc mRNA by adsorption of miRNA, leading to maintenance of high PD-L1 expression	PD-L1 combined with PD-1 on the surface of T cells significantly weakened T cell-mediated anti-tumor immune responses.
(77)	FGFR1/MAPK	Activated Brachyury protein	Upregulated PD-L1 expression inhibited the proliferation, activation, and cytotoxic function of T cells.
(78)	α5-nAChR	Promoted the phosphorylation of STAT3 and the expression of JAB1	Enhanced protein levels of PD-L1 and thus inhibited T cell-mediated anti-tumor immune responses.
(79)	SNHG12	Improved stability and increased expression levels of PD-L1 and USP8 mRNAs	Suppressed T-cell activity and enhanced immunosuppression of tumor cells, thereby promoting immune escape from NSCLC.
(80)	E2	Upregulation of PD-L1 expression through the ERβ/SIRT1 axis	Inhibits the activity of T cells, thus helping tumor cells evade recognition and attack by the immune system.
(81)	SIRPγ	Activated Hippo/YAP signaling pathway	Stimulated high CD47 expression in lung cancer cells by releasing cytokines, which consequently inhibited tumor cell phagocytosis.

SKIL, SKI Like Proto-Oncogene; TAZ, transcriptional coactivator with PDZ-binding motif; CXCL10, C-X-C motif chemokine 10; CCL5, C-C Motif Chemokine Ligand 5; STAT1, signal transducer and activator of transcription 1; TLR4, Toll-like receptor 4; IFN-β, interferon-β; TBK1, TANK-binding kinase 1; PD-1, programmed cell death protein 1; PD-L1, programmed cell death-ligand 1; PD-L2, programmed cell death-ligand 2; ERβ/SIRT1, estrogen receptors β/silent information regulator sirtuin 1; FGFR1/MARK, fibroblast growth factor receptor 1/mitogen-activated protein kinase; SIRPγ, signal regulatory protein γ; NSCLC, non-small cell lung cancer; YAP, Yes-associated protein.

## 4 Exploring novel therapeutic targets

The development of resistance or the advancement of lung cancer can be attributed to tumor cells’ ability to “stealth” within the immune system and elude detection. Therefore, it is essential to explore new therapeutic targets and strategies to enhance the effectiveness of immunotherapy and overcome resistance. As the immune escape mechanism in lung cancer has been better studied in recent years, several new targets have progressively emerged as research hotspots. The following section highlights the latest progress of these emerging targets and related clinical trials.

### 4.1 TIGIT

By attaching to its cognate ligand Poliovirus receptor (CD155), T-cell immunoglobulin and ITIM domains (TIGIT), a novel immunological checkpoint protein, can directly prevent lymphocyte activation (82). The TIGIT–PVR signaling pathway is considered a primary mechanism for tumor immune escape.

Vibostolimab (MK-7684) is a humanized IgG1 monoclonal antibody that can block the interaction of TIGIT with its ligand, restoring the anti-tumor immune response (83). The NCT02964013 study found (84) that vibostolimab was well tolerated and had preliminary anti-tumor action when applied alone or with pembrolizumab in people with advanced solid tumors. Patients with NSCLC were included in group B of the trial. This group included 39 patients who were primary anti-PD-1/PD-L1 (all receiving combination therapy) and 67 patients who were anti-PD-1/PD-L1 refractory (34 monotherapy and 33 combination treatments). In the anti-PD-1/PD-L1 primary treatment arm, the objective response rate (ORR) was 26% (n = 10/39), and 51% of patients achieved a disease control rate (DCR) (n = 20/39) and responded in both PD-L1-positive and PD-L1-negative patients. Treatment‐related adverse events were observed in 85% of nine patients, with pruritus and hypoalbuminemia being the most common. Despite a decreased overall response rate with monotherapy in anti-PD-1/PD-L1 refractory patients (3%), combination therapy demonstrated moderate efficacy (40%). However, the sample size of this trial was relatively small, which may have limited the comprehensive assessment of treatment efficacy and safety. The study had a relatively short follow-up period, which may have affected the assessment of long-term efficacy and safety. Additionally, the study did not directly compare the combination of vibostolimab and pembrolizumab with pembrolizumab monotherapy, making it difficult to accurately assess the additional benefit of combination therapy relative to single-agent therapy.

Similarly, when employed in conjunction with an anti-PD-L1 antibody, tiragolumab, a humanized monoclonal antibody, increases anticancer activity by dual immune checkpoint suppression (85). The CITYSCAPE trial (86) evaluated the effectiveness and safety of tiragolumab in combination with atezolizumab (tiragolumab arm) versus placebo in conjunction with atezolizumab (placebo arm) as a first-line treatment for PD-L1-positive NSCLC. The ORR was significantly greater in the tiragolumab group compared to the placebo group (31.3% vs. 16.2%), and the median progression-free survival (mPFS) was extended by 1.8 months in the tiragolumab group (5.4 vs. 3.6). The most common grade 3 or higher treatment-emergent adverse event (TEAE) in both groups was elevated lipase (9% vs. 3%). Overall, the combination therapy is clinically effective and has a controllable safety profile. Two phase III trials, SKYSCRAPER-01 (87) and SKYSCRAPER-02 (88), are assessing the efficacy and safety of tiragolumab in conjunction with atezolizumab in patients with advanced lung cancer based on these results. Future research needs to explore more biomarkers to better predict which patients may benefit from combination therapy. At the same time, further exploration of the optimal dosage and frequency of administration of combination therapy is available to improve treatment efficacy and reduce adverse reactions.

Moreover, rilvegostomig (AZD2936), an anti-PD-1/TIGIT bispecific agent, has entered a phase 3 clinical trial (NCT006627647). The trial was aimed to assess the efficacy and safety of rilvegostomig used along with chemotherapy as a first-line treatment for patients with non-squamous NSCLC that expresses PD-L1, with the primary objectives being overall survival (OS) and PFS. The results of this research will further verify the potential of TIGIT targets in lung cancer immunotherapy.

### 4.2 LAG-3

By attaching to MHC class II molecules or fibronectin-associated protein-1, the immunological checkpoint protein lymphocyte activation gene-3 (LAG-3) can restrict T-cell proliferation and cytokine release, resulting in immunosuppression (89, 90). Relatlimab is the first LAG-3 inhibitor approved to enhance the immune response by blocking LAG-3 interactions with its ligands (91). The Food and Drug Administration has previously approved relatlimab in association with nivolumab for the treatment of unresectable or metastatic melanoma.

With a final enrollment of 60 patients, NCT04205552 (92) investigated the safety and effectiveness of nivolumab alone (monotherapy arm) or in combination with relatlimab (combination arm) as a neoadjuvant regimen in patients with resectable NSCLC. Disease-free survival and OS were both significantly higher in the combination group than in the monotherapy group (93% and 100% vs. 89% and 93%). Tiredness, gastrointestinal issues, thyroid problems, and respiratory symptoms were the most prevalent adverse events (AEs) in both groups. Randomization in this study was not stratified according to PD-L1 status, which may have contributed to the unbalanced distribution of patients with high PD-L1 expression NSCLC in the two groups, thus affecting the assessment of the rate of deep pathological response. Moreover, the study excluded patients with extensive mediastinal lymph node metastasis, which may make the study results more optimistic than other studies of neoadjuvant ICI combination therapy in terms of surgical success rates and early survival outcomes. These results support the utilization of LAG-3 inhibitors in the treatment of lung cancer, but more research is required to determine their long-term effectiveness and optimal treatment strategies.

### 4.3 PD-1

Dostarlimab (TSR-042) blocks the interaction of PD-1 with its ligands PD-L1 and PD-L2 by specifically binding to the PD-1 receptor on the surface of T cells, restoring the ability of T cells to recognize and kill tumor cells (93). In the PERLA study (94), patients with untreated metastatic non-squamous NSCLC who received dostarlimab plus chemotherapy had a higher ORR (46% vs. 37%) and a longer mPFS (8.8 vs. 6.7 months) than those who received pembrolizumab plus chemotherapy. According to subgroup analysis, PFS was 3.7 months longer in the dostarlimab group than in the control group when PD-L1 tumor proportion score (TPS) was ≥50% (10.4 vs. 6.7) and substantially longer in the dostarlimab group when PD-L1 TPS was ≥1% (10.4 vs. 6.1 months). The most common TEAEs were anemia, fatigue, nausea, neutropenia, and vomiting.

### 4.4 TGF-β

By inhibiting T-cell activity and encouraging immunosuppressive cell infiltration in the tumor microenvironment, TGF-β, a significant immunosuppressive cytokine, assists in tumor immune evasion (95). Galunisertib (LY2157299) is a small-molecule TGF-β receptor inhibitor that prevents tumor cell proliferation, migration, and invasion by inhibiting TGF-β. Furthermore, galunisertib has the potential to counteract TGF-β-induced immunosuppression and enhance both the quantity and functionality of tumor-infiltrating CD8+ T cells, hence reinstating anticancer immune responses (96).

NCT02423343 (97) was a phase Ib/II clinical trial, with the phase II research assessing the safety of galunisertib plus nivolumab in the treatment of relapsed/refractory NSCLC patients (n = 25). According to the findings, patients with relapsed or refractory NSCLC who received galunisertib plus nivolumab achieved partial response in 24% of cases and stable disease in 16% of cases. The mPFS and OS were 5.26 and 11.99 months, respectively. The most prevalent AEs were itching, weariness, and decreased appetite. There were no AEs with a grade of ≥4. These results suggest the potential value of the TGF-β target in lung cancer immunotherapy. However, the study did not include a placebo control group, making it difficult to accurately assess the additional benefits of combination therapy relative to standard therapy. Furthermore, while the study evaluated PD-L1 expression as a biomarker, the assessment of other potential biomarkers was not comprehensive, which may limit the ability to predict treatment response.

### 4.5 CD73

Through its conversion of adenosine monophosphate to adenosine, CD73 enables the establishment of an immunosuppressive atmosphere (98). Oleclumab (MEDI9447) is a monoclonal antibody targeting CD73, which specifically binds to CD73, inhibits its enzymatic function, and diminishes the synthesis of immunosuppressive adenosine, hence augmenting the anti-tumor efficacy of immune cells (99). Monalizumab (IPH2201), a new type of immune checkpoint inhibitor, helps activate NK cells and CD8+ T cells by attaching to the NKG2A receptor and stopping it from interacting with HLA-E (100). This boosts the production of interferon-γ and makes immune cells more effective against tumors.

The COAST trial (101) assessed the efficacy of durvalumab alone (monotherapy group) or in combination with oleclumab (D+O group) or monalizumab (D+M group) in patients with unresectable phase 3 NSCLC. The results indicated that the ORR was significantly higher in the combination arm (35.0% and 40.3%) than in the single-agent arm (23.9%). The mPFS was prolonged by 8.8 months in the D+M group versus the single-agent group (15.1 vs. 6.3 months). There were no new safety signals, and all three groups had comparable safety features. In conclusion, durvalumab plus oleclumab or monalizumab significantly improved ORR and PFS in NSCLC patients compared to durvalumab monotherapy. Also, the PACIFIC-9 phase III trial is now underway to further assess the efficacy and safety of durvalumab plus oleclumab or monalizumab in a larger patient cohort (102).

### 4.6 B7-H4

The immune checkpoint ligand B7-H4 limits the growth of T cells and the production of cytokines, allowing tumor immune escape (103). SGN-B7H4V is an antibody–drug conjugate (ADC) targeting B7-H4 via a cleavable linker to the microtubule-disrupting agent, monomethyl auristatin E. In addition to its strong anticancer properties, SGN-B7H4V can activate and infiltrate immune cells.

In a preclinical trial (104), SGN-B7H4V demonstrated remarkable cytotoxicity in multiple B7-H4-positive tumor cell lines. Furthermore, when combined with the PD-1 inhibitors, SGN-B7H4V significantly increased anti-tumor activity and established long-lasting immunological memory. Toxicological results showed that SGN-B7H4V exhibited excellent tolerance in animal tests. In conclusion, SGN-B7H4V has shown significant anticancer effectiveness in preclinical models when used alone or in conjunction with PD-1 inhibitors, all while maintaining controllable safety profiles. These findings support the potential of a phase I trial (NCT05194072) to further evaluate SGN-B7H4V monotherapy or in partnership with immunotherapy.

### 4.7 TIM-3

T-cell immunoglobulin and mucin domain-containing protein 3 (TIM-3) is a negative immune checkpoint, and its high expression on T cells correlates with cellular dysfunction and tumor immunosuppression (105). Cobolimab (TSR-022/GSK4069889) is a humanized IgG4 monoclonal antibody targeting TIM-3. By blocking the binding of TIM-3 to its ligand, it maintains the binding of TIM-3 to BAT3, thus enhancing T-cell activity and restoring its anti-tumor function (106). AMBER Part 2B (NCT02817633) (107) assessed the safety and efficacy of cobolimab monotherapy or in combination with PD-1 inhibitor dostarlimab in patients with locally advanced or metastatic NSCLC. The results revealed that combination therapy demonstrated preliminary clinical activity and acceptable safety in patients with advanced/metastatic NSCLC, with an ORR of 8.3% and a DCR of 21.4%. The sample size of the research was relatively small, which may have limited the comprehensive assessment of treatment efficacy and safety. Although TIM-3 expression is a potential biomarker, its ability to predict treatment response still needs to be further validated. In addition, sabatolimab (MBG453) and LY3321367 also demonstrated corresponding clinical efficacy in the study, supporting further research into the role of the TIM-3 target in lung cancer immunotherapy.

In addition to the above therapeutic targets, CCR2/5, Galectin-3, DLL3, CD39/CD73, and NKG2A also have important research value in lung cancer immunotherapy. CCR2/5 is a key receptor for immune cell migration, and its expression in the tumor microenvironment is closely related to immune cell infiltration and function (108). By targeting CCR2/5, the distribution of immune cells can be regulated, T-cell infiltration enhanced, and the effectiveness of immunotherapy improved. Galectin-3 can bind to glycosylated ligands on the surface of tumor cells, promoting tumor cell proliferation and migration while inhibiting T-cell activity, thereby helping tumor cells evade immune surveillance (109). In immunotherapy, targeting Galectin-3 can restore T-cell activity, enhance immune responses, and thereby improve treatment efficacy. DLL3 is mainly expressed in the Golgi apparatus of SCLC cells, and some DLL3 molecules migrate to the cell surface, which can be used as a target for immunotherapy. In early-phase clinical trials (110), TIM-3 inhibitors have shown some anti-tumor activity, especially when combined with other immune checkpoint inhibitors. By targeting these molecules, it is possible to regulate immune cell infiltration in the tumor microenvironment, enhance T-cell activity, and consequently improve the efficacy of immunotherapy. **Table 2** shows the recent advances in clinical trials of ICIs for lung cancer. **Table 3** shows some ongoing clinical trials on emerging targets.

**Table 2:** Recent advances in clinical trials of ICIs for lung cancer.

Drug name	Targets	NCT number	Clinical trial	Enrollment	Phase	Primary results	Most common 3 or 4 AEs
Nivolumab (111)	PD-1	NCT04025879	CheckMate-77T	461	3	mEFS: 70.2%; pCR: 25.3%	A decreased neutrophil count.
Nivolumab (112)	PD-1	NCT02998528	CheckMate-816	358	3	mEFS: 31.6 months; pCR: 24.0%	Neutropenia, decreased neutrophil count.
Toripalimab (113)	PD-1	NCT04158440	Neotorch	501	3	2-year EFS: 64.7%; pCR: 48.5%	Anemia, neutropenia, cough, etc.
Durvalumab (114)	PD-L1	NCT03800134	AEGEAN	802	3	EFS: 73.4%; pCR: 17.2%	Neutropenia, anemia, pruritus, etc.
Pembrolizumab (115)	PD-1	NCT03425643	KEYNOTE-671	397	3	EFS: 62.4%; OS: 80.9%; pCR: 18.1%	Neutrophil count decreased, anemia, white blood cell count decreased, and platelet count decreased.
Cemiplimab (116)	PD-1	NCT03409614	EMPOWER-Lung 3	466	3	mOS: 21.9 months; mPFS: 8.2 months; ORR: 43.3%	Anemia, neutropenia.
Atezolizumab (117)	PD-L1	NCT03991403	ATLAS/KCSG-LU19-04	228	3	ORR: 69.5%; mPFS: 8.48 months	Peripheral neuropathy, alopecia, and myalgia.
Pembrolizumab (118)	PD-1	NCT03515837	KEYNOTE-789	492	3	mPFS: 5.6 months; mOS: 15.9 months	Anemia, decreased neutrophil count, nausea, etc.
Ipilimumab (119)	CTLA-4	NCT03351361	GFPC 08–2015 ENERGY	216	3	mOS: 14.7 months	Endocrine disorders, cardiac disorders, and gastrointestinal disorders.

AEs, adverse events; EFS, event-free survival; pCR, pathological complete response; OS, overall survival; PFS, progression-free survival; ORR, overall response rate.

**Table 3:** Some ongoing clinical trials on emerging targets.

NCT number	Target	Drug name	Phase	Enrollment	Primary endpoints	Secondary endpoints
NCT04623775	LAG-3	Relatlimab	2	468	TRAE, ORR	AEs, SAEs, PFS, etc.
NCT06561386	LAG-3	Relatlimab	3	1,000	OS	PFS, ORR, DOR, AEs, etc.
NCT04123379	CCR 2/5	–	2	48	MPR	PFS, OS, etc.
NCT06472076	TIGIT	Belrestotug (EOS448/GSK4428859A)	3	1,000	PFS, OS	ORR, DOR, TEAEs, etc.
NCT05565378	TIGIT	Belrestotug	2	340	ORR	PFS, OS, DOR, etc.
NCT02817633	TIM-3	TSR-022	1	447	DLT, AEs, TEAEs, etc.	DOR, ORR, DCR, etc.
NCT04931654	PD-1/TIM-3	AZD7789	1/2	136	AE, SAE, DLT	ORR, DCR, PFS, etc.
NCT03708328	PD-1/TIM-3	Lomvastomig (RO7121661)	1	134	DLT, AEs, ORR, etc.	ORR, DCR, DOR, etc.
NCT06467500	PD-1/CTLA-4	Cadonilimab (AK104)	2	48	ORR	DCR, DOR, PFS, OS, AEs.
NCT06793813	PD-1/CTLA-4	Cadonilimab	2	44	6-month PFS	–
NCT06424821	PD-1/CTLA-4	Cadonilimab	2	54	1-year PFS	OS, ORR, DOR, PFS
NCT05240131	Galectin-3	GB1211	1/2	88	Safety and tolerability	The recommended dose, Cmax, Tmax, etc.
NCT06443489	DLL3	SHR-4849	1	80	DLT, MTD, RP2D, etc.	ORR, DCR, DOR, etc.
NCT06613009	DLL3	IBI3009	1	190	DLTs, vital signs, electrocardiograms, etc.	AUC, Cmax, Tmax, etc.
NCT06424665	DLL3	FZ-AD005	1	162	DLT, MTD, AEs, etc.	PFS, DOR, OS, etc.
NCT06179069	DLL3 ADC	ZL-1310	1	112	DLTs, TEAEs, SAEs, etc.	ORR, DOR, PFS, etc.
NCT03381274	CD73	MEDI9447	1/2	43	DLTs, TEAEs	DOR, PFS, OS, etc.

TRAE, treatment-related adverse effect; ORR, overall response rate; AEs, adverse events; SAEs, serious adverse events; PFS, progression-free survival; OS, overall survival; DOR, duration of response; MPR, major pathological response; TEAEs, treatment-emergent adverse events; DLTs, dose-limiting toxicities; MTD, maximum tolerance dose; DCR, disease control rate; RP2D, recommended phase 2 dose; AUC, area under the curve; Cmax, peak concentration; Tmax, time to peak drug concentration.

## 5 Conclusion and prospect

With in-depth research into the immune escape mechanisms of lung cancer, more investigations have revealed various intrinsic and extrinsic mechanisms of tumor cells. The article outlines the various processes via which lung cancer cells elude detection and assault by the immune system, including antigen presentation defects, immune checkpoint molecule overexpression, and metabolic reprogramming, while also providing updates on contemporary therapeutic targets devised to counteract these mechanisms and the associated clinical trials.

However, considering the limitation of the number of words, the mechanism of immune escape has not been explored in depth in this article, and the interaction between the mechanisms has not been elucidated in detail. Furthermore, research on many novel targets is concentrated in the early stages of clinical trials, resulting in a low level of clinical evidence in the references. Although certain targets, including rilvegostomig, have advanced to phase III trials, immunotherapies are still confronting issues with safety, resistance, and low response rates. For instance, the ORR of TIGIT inhibitors in PD-1/PD-L1 refractory NSCLC patients was only 3%, suggesting that biomarker exploration is further needed to optimize patient stratification. The heterogeneity and dynamics of the lung cancer immune microenvironment determine the complexity of the immune escape mechanism. We believe that future research directions can be considered from the following aspects. i) Multiple targets for combination therapy. Single-target inhibition is susceptible to compensatory mechanisms. The effectiveness of the immunotherapies may be increased by combining targeted medicines, such as EGFR inhibitors plus ICIs, or by blocking several immune checkpoints at once, such as PD-1/PD-L1+TIGIT+LAG-3/CD73. For instance, the CITYSCAPE study (86) evaluated the efficacy of tiragolumab (a TIGIT inhibitor) in combination with atezolizumab (a PD-L1 inhibitor) in chemotherapy-naive patients with recurrent or metastatic NSCLC. The results indicated that the ORR in the combination therapy group was 31.3%, significantly higher than the 16.2% in the placebo plus atezolizumab group, and their median PFS was 5.4 and 3.6 months, respectively. Dual-target combination therapy offers a new treatment option for patients with low or negative PD-L1 expression who typically respond poorly to single immune checkpoint inhibitors (120). ii) Metabolism–immunity intersection studies. Research into metabolic-modifying medicines, such as Dihydrolipoamide S-acetyltransferase inhibitors, in conjunction with ICIs, has the potential to undo the tumor immunosuppressive milieu, as tumor metabolic reprogramming is intricately linked to immune escape. iii) Screening for biomarkers. The application of multi-omics detection instruments, such as single-cell sequencing, spatial transcriptomics, and metabolomics, to resolve tumor microenvironmental heterogeneity and then screen for predictive biomarkers will propel precision medicine forward. Just to illustrate, patients with low R-loop scores may benefit more from immune combination therapies. iv) Research and development of novel medications. It is anticipated that gene-editing technologies, ADCs, and bispecific monoclonal antibodies will overcome the current therapy constraints.

According to the different immune escape mechanisms of lung cancer, patients need to develop personalized treatment programs. For example, stratifying patients based on PD-L1 expression, patients with high PD-L1 expression may respond well to single immune checkpoint inhibitors, while those with low expression may require combination therapy. Based on T-cell infiltration levels, “immunologically cold” tumors with low T-cell infiltration may require the combined use of T-cell inducers. In addition to conducting a comprehensive assessment of patients prior to treatment, it is also necessary to perform dynamic monitoring during treatment and adopt appropriate combination therapy regimens based on the specific immune escape mechanisms of individual patients, thereby reducing the occurrence of drug resistance and achieving precision medicine. The potential of lung cancer immunotherapy resides in thoroughly understanding the intricate network of tumor immune interactions and advancing therapeutic options through multidisciplinary collaboration or utilizing deep learning. Immunotherapy for lung cancer is predicted to see greater advances, as basic research and clinical technology continue to advance.

## References

[1] Bray F Laversanne M Sung H Ferlay J Siegel RL Soerjomataram I. Global cancer statistics 2022: GLOBOCAN estimates of incidence and mortality worldwide for 36 cancers in 185 countries. Ca-Cancer J Clin. (2024) 74:229–63. doi: 10.3322/caac.21834, PMID: 38572751

[2] Smolarz B Lukasiewicz H Samulak D Piekarska E Kolacinski R Romanowicz H. Lung cancer-epidemiology, pathogenesis, treatment and molecular aspect (Review of literature). Int J Mol Sci. (2025) 26:2049. doi: 10.3390/ijms26052049, PMID: 40076671 PMC11900952

[3] Meyer ML Fitzgerald BG Paz-Ares L Cappuzzo F Janne PA Peters S. New promises and challenges in the treatment of advanced non-small-cell lung cancer. Lancet. (2024) 404:803–22. doi: 10.1016/S0140-6736(24)01029-8, PMID: 39121882

[4] Lin X Kang K Chen P Zeng Z Li G Xiong W. Regulatory mechanisms of PD-1/PD-L1 in cancers. Mol Cancer. (2024) 23:108. doi: 10.1186/s12943-024-02023-w, PMID: 38762484 PMC11102195

[5] Hiltbrunner S Cords L Kasser S Freiberger SN Kreutzer S Toussaint NC. Acquired resistance to anti-PD1 therapy in patients with NSCLC associates with immunosuppressive T cell phenotype. Nat Commun. (2023) 14:5154. doi: 10.1038/s41467-023-40745-5, PMID: 37620318 PMC10449840

[6] Gao M Shi J Xiao X Yao Y Chen X Wang B. PD-1 regulation in immune homeostasis and immunotherapy. Cancer Lett. (2024) 588:216726. doi: 10.1016/j.canlet.2024.216726, PMID: 38401888

[7] Tang M Xu M Wang J Liu Y Liang K Jin Y. Brain metastasis from EGFR-mutated non-small cell lung cancer: secretion of IL11 from astrocytes up-regulates PDL1 and promotes immune escape. Adv Sci. (2024) 11:e2306348. doi: 10.1002/advs.202306348, PMID: 38696655 PMC11234401

[8] Liang TL Pan HD Yan PY Mi JN Liu XC Bao WQ. Serum taurine affects lung cancer progression by regulating tumor immune escape mediated by the immune microenvironment. J Adv Res. (2024) 73:427–42. doi: 10.1016/j.jare.2024.09.005, PMID: 39243941 PMC12225919

[9] Konen JM Wu H Gibbons DL. Immune checkpoint blockade resistance in lung cancer: emerging mechanisms and therapeutic opportunities. Trends Pharmacol Sci. (2024) 45:520–36. doi: 10.1016/j.tips.2024.04.006, PMID: 38744552 PMC11189143

[10] Benvenuto M Focaccetti C. Tumor microenvironment: cellular interaction and metabolic adaptations. Int J Mol Sci. (2024) 25:3642. doi: 10.3390/ijms25073642, PMID: 38612452 PMC11011721

[11] Khosravi GR Mostafavi S Bastan S Ebrahimi N Gharibvand RS Eskandari N. Immunologic tumor microenvironment modulators for turning cold tumors hot. Cancer Commun. (2024) 44:521–53. doi: 10.1002/cac2.12539, PMID: 38551889 PMC11110955

[12] Wu J. Emerging innate immune cells in cancer immunotherapy: promises and challenges. Biodrugs. (2024) 38:499–509. doi: 10.1007/s40259-024-00657-2, PMID: 38700835 PMC11246812

[13] De Zuani M Xue H Park JS Dentro SC Seferbekova Z Tessier J. Single-cell and spatial transcriptomics analysis of non-small cell lung cancer. Nat Commun. (2024) 15:4388. doi: 10.1038/s41467-024-48700-8, PMID: 38782901 PMC11116453

[14] Onder L Papadopoulou C Lutge A Cheng HW Lutge M Perez-Shibayama C. Fibroblastic reticular cells generate protective intratumoral T cell environments in lung cancer. Cell. (2025) 188:430–46. doi: 10.1016/j.cell.2024.10.042, PMID: 39566495

[15] Wang H Liu S Zhan J Liang Y Zeng X. Shaping the immune-suppressive microenvironment on tumor-associated myeloid cells through tumor-derived exosomes. Int J Cancer. (2024) 154:2031–42. doi: 10.1002/ijc.34921, PMID: 38500385

[16] Roviello G Vascotto IA Catalano M. Editorial: Analysis of tumor immune microenvironments and molecular mechanism to reveal the dilemma of immunotherapy for advanced non-small cell lung cancer. Front Immunol. (2024) 15:1415608. doi: 10.3389/fimmu.2024.1415608, PMID: 38726009 PMC11079269

[17] Huang S Chung JY Li C Wu Y Qiao G To KF. Cellular dynamics of tumor microenvironment driving immunotherapy resistance in non-small-cell lung carcinoma. Cancer Lett. (2024) 604:217272. doi: 10.1016/j.canlet.2024.217272, PMID: 39326553

[18] Desharnais L Sorin M Rezanejad M Liu B Karimi E Atallah A. Spatially mapping the tumour immune microenvironments of non-small cell lung cancer. Nat Commun. (2025) 16:1345. doi: 10.1038/s41467-025-56546-x, PMID: 39905080 PMC11794701

[19] Zhang X Zhang Z Liu Y Zhao S Zhao X Zhang L. Investigating lung cancer microenvironment from cell segmentation of pathological image and its application in prognostic stratification. Sci Rep-Uk. (2025) 15:1704. doi: 10.1038/s41598-025-85532-y, PMID: 39799232 PMC11724888

[20] Lim JU Lee E Lee S Cho HJ Ahn DH Hwang Y. Current literature review on the tumor immune micro-environment, its heterogeneity and future perspectives in treatment of advanced non-small cell lung cancer. Transl Lung Cancer R. (2023) 12:857–76. doi: 10.21037/tlcr-22-633, PMID: 37197639 PMC10183402

[21] Dunn GP Bruce AT Ikeda H Old LJ Schreiber RD. Cancer immunoediting: from immunosurveillance to tumor escape. Nat Immunol. (2002) 3:991–8. doi: 10.1038/ni1102-991, PMID: 12407406

[22] Galassi C Chan TA Vitale I Galluzzi L. The hallmarks of cancer immune evasion. Cancer Cell. (2024) 42:1825–63. doi: 10.1016/j.ccell.2024.09.010, PMID: 39393356

[23] Yan Y Sun D Hu J Chen Y Sun L Yu H. Multi-omic profiling highlights factors associated with resistance to immuno-chemotherapy in non-small-cell lung cancer. Nat Genet. (2025) 57:126–39. doi: 10.1038/s41588-024-01998-y, PMID: 39658657

[24] Wang Y Jasinski-Bergner S Wickenhauser C Seliger B. Cancer immunology: immune escape of tumors-expression and regulation of HLA class I molecules and its role in immunotherapies. Adv Anat Pathol. (2023) 30:148–59. doi: 10.1097/PAP.0000000000000389, PMID: 36517481

[25] Montesion M Murugesan K Jin DX Sharaf R Sanchez N Guria A. Somatic HLA class I loss is a widespread mechanism of immune evasion which refines the use of tumor mutational burden as a biomarker of checkpoint inibitor response. Cancer Discov. (2021) 11:282–92. doi: 10.1158/2159-8290.CD-20-0672, PMID: 33127846

[26] Roerden M Castro AB Cui Y Harake N Kim B Dye J. Neoantigen architectures define immunogenicity and drive immune evasion of tumors with heterogenous neoantigen expression. J Immunother Cancer. (2024) 12:e010249. doi: 10.1136/jitc-2024-010249, PMID: 39521615 PMC11552027

[27] Hu Z Guo X Li Z Meng Z Huang S. The neoantigens derived from transposable elements - A hidden treasure for cancer immunotherapy. Bba-Rev Cancer. (2024) 1879:189126. doi: 10.1016/j.bbcan.2024.189126, PMID: 38849060

[28] Zhou F Guo H Xia Y Le X Tan D Ramalingam SS. The changing treatment landscape of EGFR-mutant non-small-cell lung cancer. Nat Rev Clin Oncol. (2025) 22:95–116. doi: 10.1038/s41571-024-00971-2, PMID: 39614090

[29] Bian HT Shen YW Zhou YD Nagle DG Guan YY Zhang WD. CD47: Beyond an immune checkpoint in cancer treatment. Bba-Rev Cancer. (2022) 1877:188771. doi: 10.1016/j.bbcan.2022.188771, PMID: 35931392

[30] Hu LY Zhuang WT Chen MJ Liao J Wu DF Zhang YX. EGFR oncogenic mutations in NSCLC impair macrophage phagocytosis and mediate innate immune evasion through up-regulation of CD47. J Thorac Oncol. (2024) 19:1186–200. doi: 10.1016/j.jtho.2024.03.019, PMID: 38553005

[31] De Leo A Ugolini A Yu X Scirocchi F Scocozza D Peixoto B. Glucose-driven histone lactylation promotes the immunosuppressive activity of monocyte-derived macrophages in glioblastoma. Immunity. (2024) 57:1105–23. doi: 10.1016/j.immuni.2024.04.006, PMID: 38703775 PMC11114377

[32] Zhang C Zhou L Zhang M Du Y Li C Ren H. H3K18 lactylation potentiates immune escape of non-small cell lung cancer. Cancer Res. (2024) 84:3589–601. doi: 10.1158/0008-5472.CAN-23-3513, PMID: 39137401

[33] Miller KD O’Connor S Pniewski KA Kannan T Acosta R Mirji G. Acetate acts as a metabolic immunomodulator by bolstering T-cell effector function and potentiating antitumor immunity in breast cancer. Nat Cancer. (2023) 4:1491–507. doi: 10.1038/s43018-023-00636-6, PMID: 37723305 PMC10615731

[34] Wang J Yang Y Shao F Meng Y Guo D He J. Acetate reprogrammes tumour metabolism and promotes PD-L1 expression and immune evasion by upregulating c-Myc. Nat Metab. (2024) 6:914–32. doi: 10.1038/s42255-024-01037-4, PMID: 38702440

[35] Zhang H Li S Wang D Liu S Xiao T Gu W. Metabolic reprogramming and immune evasion: the interplay in the tumor microenvironment. biomark Res. (2024) 12:96. doi: 10.1186/s40364-024-00646-1, PMID: 39227970 PMC11373140

[36] DePeaux K Delgoffe GM. Metabolic barriers to cancer immunotherapy. Nat Rev Immunol. (2021) 21:785–97. doi: 10.1038/s41577-021-00541-y, PMID: 33927375 PMC8553800

[37] Yuan Y Ju YS Kim Y Li J Wang Y Yoon CJ. Author Correction: Comprehensive molecular characterization of mitochondrial genomes in human cancers. Nat Genet. (2023) 55:1078. doi: 10.1038/s41588-023-01317-x, PMID: 36944732 PMC10260393

[38] Gorelick AN Kim M Chatila WK La K Hakimi AA Berger MF. Respiratory complex and tissue lineage drive recurrent mutations in tumour mtDNA. Nat Metab. (2021) 3:558–70. doi: 10.1038/s42255-021-00378-8, PMID: 33833465 PMC9304985

[39] Ikeda H Kawase K Nishi T Watanabe T Takenaga K Inozume T. Immune evasion through mitochondrial transfer in the tumour microenvironment. Nature. (2025) 638:225–36. doi: 10.1038/s41586-024-08439-0, PMID: 39843734 PMC11798832

[40] Elsakrmy N Cui H. R-loops and R-loop-binding proteins in cancer progression and drug resistance. Int J Mol Sci. (2023) 24:7064. doi: 10.3390/ijms24087064, PMID: 37108225 PMC10138518

[41] Petermann E Lan L Zou L. Sources, resolution and physiological relevance of R-loops and RNA-DNA hybrids. Nat Rev Mol Cell Bio. (2022) 23:521–40. doi: 10.1038/s41580-022-00474-x, PMID: 35459910

[42] Zhang S Liu Y Sun Y Liu Q Gu Y Huang Y. Aberrant R-loop-mediated immune evasion, cellular communication, and metabolic reprogramming affect cancer progression: a single-cell analysis. Mol Cancer. (2024) 23:11. doi: 10.1186/s12943-023-01924-6, PMID: 38200551 PMC10777569

[43] Lee SY Kwak MJ Kim JJ. R-loops: a key driver of inflammatory responses in cancer. Exp Mol Med. (2025) 57:1455–66. doi: 10.1038/s12276-025-01495-0, PMID: 40629041 PMC12322051

[44] Norris K Hopes T Aspden JL. Ribosome heterogeneity and specialization in development. Wires RNA. (2021) 12:e1644. doi: 10.1002/wrna.1644, PMID: 33565275 PMC8647923

[45] Dopler A Alkan F Malka Y van der Kammen R Hoefakker K Taranto D. P-stalk ribosomes act as master regulators of cytokine-mediated processes. Cell. (2024) 187:6981–6993.e23. doi: 10.1016/j.cell.2024.09.039, PMID: 39437780 PMC11896023

[46] Li Y Li Z Tang Y Zhuang X Feng W Boor P. Unlocking the therapeutic potential of the NKG2A-HLA-E immune checkpoint pathway in T cells and NK cells for cancer immunotherapy. J Immunother Cancer. (2024) 12:e009934. doi: 10.1136/jitc-2024-009934, PMID: 39486805 PMC11529472

[47] De Wispelaere W Annibali D Tuyaerts S Messiaen J Antoranz A Shankar G. PI3K/mTOR inhibition induces tumour microenvironment remodelling and sensitises pS6(high) uterine leiomyosarcoma to PD-1 blockade. Clin Transl Med. (2024) 14:e1655. doi: 10.1002/ctm2.1655, PMID: 38711203 PMC11074386

[48] Chang W Luo Q Wu X Nan Y Zhao P Zhang L. OTUB2 exerts tumor-suppressive roles via STAT1-mediated CALML3 activation and increased phosphatidylserine synthesis. Cell Rep. (2022) 41:111561. doi: 10.1016/j.celrep.2022.111561, PMID: 36288705

[49] Ren W Xu Z Chang Y Ju F Wu H Liang Z. Pharmaceutical targeting of OTUB2 sensitizes tumors to cytotoxic T cells via degradation of PD-L1. Nat Commun. (2024) 15:9. doi: 10.1038/s41467-023-44466-7, PMID: 38167274 PMC10761827

[50] Chen Y Yang S Zhou H Su D. PRDX2 promotes the proliferation and metastasis of non-small cell lung cancer *in vitro* and *in vivo* . BioMed Res Int. (2020) 2020:8359860. doi: 10.1155/2020/8359860, PMID: 32908916 PMC7474358

[51] Zheng S Song J Linghu D Yang R Liu B Xue Z. Galectin-9 blockade synergizes with ATM inhibition to induce potent anti-tumor immunity. Int J Biol Sci. (2023) 19:981–93. doi: 10.7150/ijbs.79852, PMID: 36778120 PMC9909994

[52] Dong Y Cheng A Zhou J Guo J Liu Y Li X. PRDX2 induces tumor immune evasion by modulating the HDAC3-Galectin-9 axis in lung adenocarcinoma cells. J Transl Med. (2025) 23:81. doi: 10.1186/s12967-024-05888-z, PMID: 39825365 PMC11740609

[53] Xiao B Li G Gulizeba H Liu H Sima X Zhou T. Choline metabolism reprogramming mediates an immunosuppressive microenvironment in non-small cell lung cancer (NSCLC) by promoting tumor-associated macrophage functional polarization and endothelial cell proliferation. J Transl Med. (2024) 22:442. doi: 10.1186/s12967-024-05242-3, PMID: 38730286 PMC11084143

[54] Scafetta G D’Alessandria C Bartolazzi A. Galectin-3 and cancer immunotherapy: a glycobiological rationale to overcome tumor immune escape. J Exp Clin Canc Res. (2024) 43:41. doi: 10.1186/s13046-024-02968-2, PMID: 38317202 PMC10845537

[55] Unal B Kuzu OF Jin Y Osorio D Kildal W Pradhan M. Targeting IRE1alpha reprograms the tumor microenvironment and enhances anti-tumor immunity in prostate cancer. Nat Commun. (2024) 15:8895. doi: 10.1038/s41467-024-53039-1, PMID: 39406723 PMC11480464

[56] Crowley M Bhinder B Markowitz GJ Martin M Verma A Sandoval TA. Tumor-intrinsic IRE1alpha signaling controls protective immunity in lung cancer. Nat Commun. (2023) 14:120. doi: 10.1038/s41467-022-35584-9, PMID: 36624093 PMC9829901

[57] Nian Z Dou Y Shen Y Liu J Du X Jiang Y. Interleukin-34-orchestrated tumor-associated macrophage reprogramming is required for tumor immune escape driven by p53 inactivation. Immunity. (2024) 57:2344–61. doi: 10.1016/j.immuni.2024.08.015, PMID: 39321806

[58] Di Y Wang Z Xiao J Zhang X Ye L Wen X. ACSL6-activated IL-18R1-NF-kappaB promotes IL-18-mediated tumor immune evasion and tumor progression. Sci Adv. (2024) 10:eadp0719. doi: 10.1126/sciadv.adp0719, PMID: 39292786 PMC11409972

[59] Heim L Yang Z Tausche P Hohenberger K Chiriac MT Koelle J. IL-9 producing tumor-infiltrating lymphocytes and treg subsets drive immune escape of tumor cells in non-small cell lung cancer. Front Immunol. (2022) 13:859738. doi: 10.3389/fimmu.2022.859738, PMID: 35514957 PMC9065342

[60] Chen R Yang X Liu Q Zhang S Ma L. Research progresses on the effects of CCL4 on immune escape in the tumor microenvironment. Zhongguo Fei Ai Za Zhi. (2024) 27:613–21. doi: 10.3779/j.issn.1009-3419.2024.106.23, PMID: 39318254 PMC11425676

[61] Mukaida N Sasaki SI Baba T. CCL4 signaling in the tumor microenvironment. Adv Exp Med Biol. (2020) 1231:23–32. doi: 10.1007/978-3-030-36667-4_3, PMID: 32060843

[62] Chen R Ma L Jiang C Zhang S. Expression and potential role of CCL4 in CD8+T cells in NSCLC. Clin Transl Oncol. (2022) 24:2420–31. doi: 10.1007/s12094-022-02913-9, PMID: 35964269

[63] Luo H Hu B Gu XR Chen J Fan XQ Zhang W. The miR-23a/27a/24–2 cluster drives immune evasion and resistance to PD-1/PD-L1 blockade in non-small cell lung cancer. Mol Cancer. (2024) 23:285. doi: 10.1186/s12943-024-02201-w, PMID: 39736629 PMC11686834

[64] Hou J Wang Z Yang L Guo X Yang G. The function of EMSY in cancer development. Tumour Biol. (2014) 35:5061–6. doi: 10.1007/s13277-013-1584-3, PMID: 24609898

[65] Marzio A Kurz E Sahni JM Di Feo G Puccini J Jiang S. EMSY inhibits homologous recombination repair and the interferon response, promoting lung cancer immune evasion. Cell. (2022) 185:169–83. doi: 10.1016/j.cell.2021.12.005, PMID: 34963055 PMC8751279

[66] Yu Q Peng X Xu G Bai X Cao Y Du Y. Overexpression or knockdown of the P2X7 receptor regulates the progression of non-small cell lung cancer, involving GSK-3beta and JNK signaling pathways. Eur J Pharmacol. (2025) 995:177421. doi: 10.1016/j.ejphar.2025.177421, PMID: 39993700

[67] Zou YT Li JY Chai JY Hu YS Zhang WJ Zhang Q. The impact of the P2X7 receptor on the tumor immune microenvironment and its effects on tumor progression. Biochem Bioph Res Co. (2024) 707:149513. doi: 10.1016/j.bbrc.2024.149513, PMID: 38508051

[68] Sainz RM Rodriguez-Quintero JH Maldifassi MC Stiles BM Wennerberg E. Tumour immune escape via P2X7 receptor signalling. Front Immunol. (2023) 14:1287310. doi: 10.3389/fimmu.2023.1287310, PMID: 38022596 PMC10643160

[69] Adinolfi E De Marchi E Grignolo M Szymczak B Pegoraro A. The P2X7 receptor in oncogenesis and metastatic dissemination: new insights on vesicular release and adenosinergic crosstalk. Int J Mol Sci. (2023) 24:13906. doi: 10.3390/ijms241813906, PMID: 37762206 PMC10531279

[70] Ma F Ding MG Lei YY Luo LH Jiang S Feng YH. SKIL facilitates tumorigenesis and immune escape of NSCLC via upregulating the TAZ/autophagy axis. Cell Death Dis. (2020) 11:1028. doi: 10.1038/s41419-020-03200-7, PMID: 33268765 PMC7710697

[71] Chen Y Xu J Wu X Yao H Yan Z Guo T. CD147 regulates antitumor CD8(+) T-cell responses to facilitate tumor-immune escape. Cell Mol Immunol. (2021) 18:1995–2009. doi: 10.1038/s41423-020-00570-y, PMID: 33177695 PMC8322173

[72] Breitenecker K Homolya M Luca AC Lang V Trenk C Petroczi G. Down-regulation of A20 promotes immune escape of lung adenocarcinomas. Sci Transl Med. (2021) 13:eabc3911. doi: 10.1126/scitranslmed.abc3911, PMID: 34233950 PMC7611502

[73] Wang K Wang J Liu T Yu W Dong N Zhang C. Morphine-3-glucuronide upregulates PD-L1 expression via TLR4 and promotes the immune escape of non-small cell lung cancer. Cancer Biol Med. (2021) 18:155–71. doi: 10.20892/j.issn.2095-3941.2020.0442, PMID: 33628591 PMC7877184

[74] Xie M Fu X Jiang K. Notch1/TAZ axis promotes aerobic glycolysis and immune escape in lung cancer. Cell Death Dis. (2021) 12:832. doi: 10.1038/s41419-021-04124-6, PMID: 34482375 PMC8418606

[75] Jiang C Zhang N Hu X Wang H. Tumor-associated exosomes promote lung cancer metastasis through multiple mechanisms. Mol Cancer. (2021) 20:117. doi: 10.1186/s12943-021-01411-w, PMID: 34511114 PMC8436438

[76] Xia R Geng G Yu X Xu Z Guo J Liu H. LINC01140 promotes the progression and tumor immune escape in lung cancer by sponging multiple microRNAs. J Immunother Cancer. (2021) 9:e002746. doi: 10.1136/jitc-2021-002746, PMID: 34446576 PMC8395365

[77] Hu Y Lu Y Xing F Hsu W. FGFR1/MAPK-directed brachyury activation drives PD-L1-mediated immune evasion to promote lung cancer progression. Cancer Lett. (2022) 547:215867. doi: 10.1016/j.canlet.2022.215867, PMID: 35985510

[78] Zhu P Jin Z Kang G Jia Y Liu D Zhang Q. Alpha5 nicotinic acetylcholine receptor-mediated immune escape of lung adenocarcinoma via STAT3/Jab1-PD-L1 signalling. Cell Commun Signal. (2022) 20:121. doi: 10.1186/s12964-022-00934-z, PMID: 35971127 PMC9377093

[79] Huang Y Xia L Tan X Zhang J Zeng W Tan B. Molecular mechanism of lncRNA SNHG12 in immune escape of non-small cell lung cancer through the HuR/PD-L1/USP8 axis. Cell Mol Biol Lett. (2022) 27:43. doi: 10.1186/s11658-022-00343-7, PMID: 35658874 PMC9164758

[80] Song S Tang H Quan W Shang A Ling C. Estradiol initiates the immune escape of non-small cell lung cancer cells via ERbeta/SIRT1/FOXO3a/PD-L1 axis. Int Immunopharmacol. (2022) 107:108629. doi: 10.1016/j.intimp.2022.108629, PMID: 35344811

[81] Xu C Jin G Wu H Cui W Wang YH Manne RK. SIRPgamma-expressing cancer stem-like cells promote immune escape of lung cancer via Hippo signaling. J Clin Invest. (2022) 132:e141797. doi: 10.1172/JCI141797, PMID: 35229723 PMC8884909

[82] Liu Y Liu W Wu T. TIGIT: Will it be the next star therapeutic target like PD-1 in hematological Malignancies? Crit Rev Oncol Hemat. (2024) 204:104495. doi: 10.1016/j.critrevonc.2024.104495, PMID: 39236904

[83] An anti-TIGIT antibody with a PD-1 inhibitor shows promise in solid tumors. Cancer Discov. (2022) 12:14. doi: 10.1158/2159-8290.CD-RW2021-170, PMID: 34819317

[84] Niu J Maurice-Dror C Lee DH Kim DW Nagrial A Voskoboynik M. First-in-human phase 1 study of the anti-TIGIT antibody vibostolimab as monotherapy or with pembrolizumab for advanced solid tumors, including non-small-cell lung cancer. Ann Oncol. (2022) 33:169–80. doi: 10.1016/j.annonc.2021.11.002, PMID: 34800678

[85] Garralda E Oh DY Italiano A Bedard PL Delord JP Calvo E. Pharmacokinetics (PK) of tiragolumab in first-in-human study in patients with mixed solid tumors (GO30103). J Clin Pharmacol. (2024) 64:544–54. doi: 10.1002/jcph.2397, PMID: 38105505

[86] Cho BC Abreu DR Hussein M Cobo M Patel AJ Secen N. Tiragolumab plus atezolizumab versus placebo plus atezolizumab as a first-line treatment for PD-L1-selected non-small-cell lung cancer (CITYSCAPE): primary and follow-up analyses of a randomised, double-blind, phase 2 study. Lancet Oncol. (2022) 23:781–92. doi: 10.1016/S1470-2045(22)00226-1, PMID: 35576957

[87] Cortiula F Reymen B Peters S Van Mol P Wauters E Vansteenkiste J. Immunotherapy in unresectable stage III non-small-cell lung cancer: state of the art and novel therapeutic approaches. Ann Oncol. (2022) 33:893–908. doi: 10.1016/j.annonc.2022.06.013, PMID: 35777706

[88] Rudin CM Liu SV Soo RA Lu S Hong MH Lee JS. SKYSCRAPER-02: tiragolumab in combination with atezolizumab plus chemotherapy in untreated extensive-stage small-cell lung cancer. J Clin Oncol. (2024) 42:324–35. doi: 10.1200/JCO.23.01363, PMID: 37976444 PMC10824371

[89] Huo JL Wang YT Fu WJ Lu N Liu ZS. The promising immune checkpoint LAG-3 in cancer immunotherapy: from basic research to clinical application. Front Immunol. (2022) 13:956090. doi: 10.3389/fimmu.2022.956090, PMID: 35958563 PMC9361790

[90] Shi AP Tang XY Xiong YL Zheng KF Liu YJ Shi XG. Immune checkpoint LAG3 and its ligand FGL1 in cancer. Front Immunol. (2021) 12:785091. doi: 10.3389/fimmu.2021.785091, PMID: 35111155 PMC8801495

[91] Su J Fu Y Cui Z Abidin Z Yuan J Zhang X. Relatlimab: a novel drug targeting immune checkpoint LAG-3 in melanoma therapy. Front Pharmacol. (2023) 14:1349081. doi: 10.3389/fphar.2023.1349081, PMID: 38269271 PMC10806167

[92] Schuler M Cuppens K Plones T Wiesweg M Du Pont B Hegedus B. Neoadjuvant nivolumab with or without relatlimab in resectable non-small-cell lung cancer: a randomized phase 2 trial. Nat Med. (2024) 30:1602–11. doi: 10.1038/s41591-024-02965-0, PMID: 38689060 PMC11186754

[93] Markham A. Dostarlimab: first approval. DRUGS. (2021) 81:1213–9. doi: 10.1007/s40265-021-01539-5, PMID: 34106455

[94] Lim SM Peters S Ortega GA Pinto G Fuentes CS Lo RG. Dostarlimab or pembrolizumab plus chemotherapy in previously untreated metastatic non-squamous non-small cell lung cancer: the randomized PERLA phase II trial. Nat Commun. (2023) 14:7301. doi: 10.1038/s41467-023-42900-4, PMID: 37951954 PMC10640551

[95] Schmidt D Endres C Hoefflin R Andrieux G Zwick M Karantzelis N. Oncogenic calreticulin induces immune escape by stimulating TGFbeta expression and regulatory T-cell expansion in the bone marrow microenvironment. Cancer Res. (2024) 84:2985–3003. doi: 10.1158/0008-5472.CAN-23-3553, PMID: 38885318 PMC11405138

[96] Tschernia NP Gulley JL. Tumor in the crossfire: inhibiting TGF-beta to enhance cancer immunotherapy. Biodrugs. (2022) 36:153–80. doi: 10.1007/s40259-022-00521-1, PMID: 35353346 PMC8986721

[97] Nadal E Saleh M Aix SP Ochoa-de-Olza M Patel SP Antonia S. A phase Ib/II study of galunisertib in combination with nivolumab in solid tumors and non-small cell lung cancer. BMC Cancer. (2023) 23:708. doi: 10.1186/s12885-023-11153-1, PMID: 37507657 PMC10386782

[98] Kowash RR Akbay EA. Tumor intrinsic and extrinsic functions of CD73 and the adenosine pathway in lung cancer. Front Immunol. (2023) 14:1130358. doi: 10.3389/fimmu.2023.1130358, PMID: 37033953 PMC10079876

[99] Bendell J LoRusso P Overman M Noonan AM Kim DW Strickler JH. First-in-human study of oleclumab, a potent, selective anti-CD73 monoclonal antibody, alone or in combination with durvalumab in patients with advanced solid tumors. Cancer Immunol Immun. (2023) 72:2443–58. doi: 10.1007/s00262-023-03430-6, PMID: 37016126 PMC10264501

[100] Patel SP Alonso-Gordoa T Banerjee S Wang D Naidoo J Standifer NE. Phase 1/2 study of monalizumab plus durvalumab in patients with advanced solid tumors. J Immunother Cancer. (2024) 12:e007340. doi: 10.1136/jitc-2023-007340, PMID: 38309722 PMC10840023

[101] Herbst RS Majem M Barlesi F Carcereny E Chu Q Monnet I. COAST: an open-label, phase II, multidrug platform study of durvalumab alone or in combination with oleclumab or monalizumab in patients with unresectable, stage III non-small-cell lung cancer. J Clin Oncol. (2022) 40:3383–93. doi: 10.1200/JCO.22.00227, PMID: 35452273

[102] Barlesi F Cho BC Goldberg SB Yoh K Zimmer GA Mann H. PACIFIC-9: Phase III trial of durvalumab + oleclumab or monalizumab in unresectable stage III non-small-cell lung cancer. Future Oncol. (2024) 20:2137–47. doi: 10.1080/14796694.2024.2354160, PMID: 39023287 PMC11508940

[103] Gitto SB Whicker M Davies G Kumar S Kinneer K Xu H. A B7-H4-targeting antibody-drug conjugate shows antitumor activity in PARPi and platinum-resistant cancers with B7-H4 expression. Clin Cancer Res. (2024) 30:1567–81. doi: 10.1158/1078-0432.CCR-23-1079, PMID: 37882675 PMC11034955

[104] Gray E Ulrich M Epp A Younan P Sahetya D Hensley K. SGN-B7H4V, an investigational vedotin ADC directed to the immune checkpoint ligand B7-H4, shows promising activity in preclinical models. J Immunother Cancer. (2023) 11:e007572. doi: 10.1136/jitc-2023-007572, PMID: 37793853 PMC10551938

[105] Dixon KO Lahore GF Kuchroo VK. Beyond T cell exhaustion: TIM-3 regulation of myeloid cells. Sci Immunol. (2024) 9:eadf2223. doi: 10.1126/sciimmunol.adf2223, PMID: 38457514

[106] Gomes De Morais AL Cerda S de Miguel M. New checkpoint inhibitors on the road: targeting TIM-3 in solid tumors. Curr Oncol Rep. (2022) 24:651–8. doi: 10.1007/s11912-022-01218-y, PMID: 35218498

[107] Davar D Eroglu Z Perez CL Di Pace B Wang T Yanamandra N. Combined targeting of PD-1 and TIM-3 in patients with locally advanced or metastatic melanoma: AMBER cohorts 1c, 1e, and 2A. Clin Cancer Res. (2025) 31:3433–42. doi: 10.1158/1078-0432.CCR-25-0884, PMID: 40552935 PMC12351273

[108] Ding J Yeong C. Advances in DLL3-targeted therapies for small cell lung cancer: challenges, opportunities, and future directions. Front Oncol. (2024) 14:1504139. doi: 10.3389/fonc.2024.1504139, PMID: 39703856 PMC11655346

[109] Wang Q Wu Y Jiang G Huang X. Galectin-3 induces pathogenic immunosuppressive macrophages through interaction with TREM2 in lung cancer. J Exp Clin Canc Res. (2024) 43:224. doi: 10.1186/s13046-024-03124-6, PMID: 39135069 PMC11321020

[110] Roussot N Kaderbhai C Ghiringhelli F. Targeting immune checkpoint inhibitors for non-small-cell lung cancer: beyond PD-1/PD-L1 monoclonal antibodies. CancerS. (2025) 17:906. doi: 10.3390/cancers17050906, PMID: 40075753 PMC11898530

[111] Cascone T Awad MM Spicer JD He J Lu S Sepesi B. Perioperative nivolumab in resectable lung cancer. New Engl J Med. (2024) 390:1756–69. doi: 10.1056/NEJMoa2311926, PMID: 38749033

[112] Forde PM Spicer J Lu S Provencio M Mitsudomi T Awad MM. Neoadjuvant nivolumab plus chemotherapy in resectable lung cancer. New Engl J Med. (2022) 386:1973–85. doi: 10.1056/NEJMoa2202170, PMID: 35403841 PMC9844511

[113] Lu S Zhang W Wu L Wang W Zhang P Fang W. Perioperative toripalimab plus chemotherapy for patients with resectable non-small cell lung cancer: the neotorch randomized clinical trial. Jama-J Am Med Assoc. (2024) 331:201–11. doi: 10.1001/jama.2023.24735, PMID: 38227033 PMC10792477

[114] Horita N Fujiwara Y. Perioperative durvalumab for resectable non-small-cell lung cancer. New Engl J Med. (2024) 390:287. doi: 10.1056/NEJMc2313778, PMID: 38231634

[115] Spicer JD Garassino MC Wakelee H Liberman M Kato T Tsuboi M. Neoadjuvant pembrolizumab plus chemotherapy followed by adjuvant pembrolizumab compared with neoadjuvant chemotherapy alone in patients with early-stage non-small-cell lung cancer (KEYNOTE-671): a randomised, double-blind, placebo-controlled, phase 3 trial. Lancet. (2024) 404:1240–52. doi: 10.1016/S0140-6736(24)01756-2, PMID: 39288781 PMC11512588

[116] Gogishvili M Melkadze T Makharadze T Giorgadze D Dvorkin M Penkov K. Cemiplimab plus chemotherapy versus chemotherapy alone in non-small cell lung cancer: a randomized, controlled, double-blind phase 3 trial. Nat Med. (2022) 28:2374–80. doi: 10.1038/s41591-022-01977-y, PMID: 36008722 PMC9671806

[117] Park S Kim TM Han JY Lee GW Shim BY Lee YG. Randomized study of atezolizumab plus bevacizumab and chemotherapy in patients with EGFR- or ALK-mutated non-small-cell lung cancer (ATTLAS, KCSG-LU19-04). J Clin Oncol. (2024) 42:1241–51. doi: 10.1200/JCO.23.01891, PMID: 37861993 PMC11095857

[118] Yang JC Lee DH Lee JS Fan Y de Marinis F Iwama E. Phase III KEYNOTE-789 study of pemetrexed and platinum with or without pembrolizumab for tyrosine kinase inhibitor–Resistant, EGFR-mutant, metastatic nonsquamous non-small cell lung cancer. J Clin Oncol. (2024) 42:4029–39. doi: 10.1200/JCO.23.02747, PMID: 39173098 PMC11608596

[119] Lena H Greillier L Cropet C Bylicki O Monnet I Audigier-Valette C. Nivolumab plus ipilimumab versus carboplatin-based doublet as first-line treatment for patients with advanced non-small-cell lung cancer aged >/=70 years or with an ECOG performance status of 2 (GFPC 08–2015 ENERGY): a randomised, open-label, phase 3 study. Lancet Resp Med. (2025) 13:141–52. doi: 10.1016/S2213-2600(24)00264-9, PMID: 39486424

[120] Guan X Hu R Choi Y Srivats S Nabet BY Silva J. Anti-TIGIT antibody improves PD-L1 blockade through myeloid and T(reg) cells. Nature. (2024) 627:646–55. doi: 10.1038/s41586-024-07121-9, PMID: 38418879 PMC11139643

